# Metabolome analysis reveals flavonoid changes during the leaf color transition in *Populus × euramericana* ‘Zhonghuahongye’

**DOI:** 10.3389/fpls.2023.1162893

**Published:** 2023-05-08

**Authors:** Mengjiao Chen, Cuifang Chang, Hui Li, Lin Huang, Zongshun Zhou, Jingle Zhu, Dan Liu

**Affiliations:** ^1^ Research Institute of Non-Timber Forestry, Chinese Academy of Forestry, Zhengzhou, Henan, China; ^2^ Research Institute of Tropical Forestry, Chinese Academy of Forestry, Guangzhou, Guangdong, China; ^3^ College of Forestry, Henan Agricultural University, Zhengzhou, Henan, China; ^4^ China Experimental Centre of Subtropical Forestry, Chinese Academy of Forestry, Xinyu, Jiangxi, China; ^5^ Shandong Provincial Center of Forest and Grass Germplasm Resources, Jinan, Shandong, China

**Keywords:** color change, metabolome analysis, flavonoids, Poplar ‘Zhonghong’, leaves

## Abstract

**Introduction:**

To investigate the mechanism of leaf color change at different stages in *Populus × euramericana* ‘Zhonghuahongye’ (‘Zhonghong’ poplar).

**Methods:**

Leaf color phenotypes were determined and a metabolomic analysis was performed on leaves at three stages (R1, R2 and R3).

**Results:**

The a*, C* and chromatic light values of the leaves decreased by 108.91%, 52.08% and 113.34%, while the brightness *L* values and chromatic b* values gradually increased by 36.01% and 13.94%, respectively. In the differential metabolite assay, 81 differentially expressed metabolites were detected in the R1 vs. R3 comparison, 45 were detected in the R1 vs. R2 comparison, and 75 were detected in the R2 vs. R3 comparison. Ten metabolites showed significant differences in all comparisons, which were mostly flavonoid metabolites. The metabolites that were upregulated in the three periods were cyanidin 3,5-O-diglucoside, delphinidin, and gallocatechin, with flavonoid metabolites accounting for the largest proportion and malvidin 3- O-galactoside as the primary downregulated metabolite. The color shift of red leaves from a bright purplish red to a brownish green was associated with the downregulation of malvidin 3-O-glucoside, cyanidin, naringenin, and dihydromyricetin.

**Discussion:**

Here, we analyzed the expression of flavonoid metabolites in the leaves of ‘Zhonghong’ poplar at three stages and identified key metabolites closely related to leaf color change, providing an important genetic basis for the genetic improvement of this cultivar.

## 1 Introduction

The polymorphic color of plant leaves is mainly determined by a combination of pigments: chlorophylls (Chl a and Chl b), carotenoids (lutein and carotenoids) and flavonoids (anthocyanins) (Cheng et al., 2015). Chlorophylls (green), carotenoids (from yellow to red) and anthocyanins (from red to purple) contribute greatly to the color presentation of leaves (Zhang et al., 2020). Among the many pigments, anthocyanins are of wide interest because of their considerable ability to control the color change of leaves (Zhao et al., 2020).

Anthocyanins are water-soluble natural pigments that are widely found in plants and are a type of flavonoid (Dooner et al., 1991; Jeong et al., 2004). Anthocyanins are synthesized by the flavonoid biosynthetic pathway and are subsequently transported into the vacuoles. Structurally, flavonoids consist of a central three-carbon chain (C6-C3-C6) linked to two aromatic rings (A and B) (Falcone-Ferreyra et al., 2012). Based on the modification of the heterocycle with three carbon atoms in the center, flavonoids are classified as flavanones, flavones, flavanols, dihydroflavonols, isoflavones and anthocyanins (Zhang and Hao, 2020). Anthocyanins are flavonoids with a special molecular structure and high water solubility. Depending on their molecular structure, anthocyanins are classified into six classes: malvidin, petunidin, pelargonidin, peonidin, delphinidin and cyanidin. They are distinguished according to the hydroxyl pattern or methoxy substitution of the aromatic B ring. Malvidin, delphinidin and peonidin mainly cause purple and dark colors, while cyanidin and pelargonidin and their derivatives mainly cause bright red colors (Jaakola, 2013; Cho et al., 2016).

Populus is the dominant tree genus in northern China, playing an important role in urban and rural greening, wind and sand control and the timber industry (Carle, 2005). The poplar cultivar ‘Zhonghong’ is obtained from a bud mutation of ‘L2025’ poplar (a green-leaved poplar) with red leaves and significant seasonal phenotype changes and has a striking effect in landscape aesthetics (Tian et al., 2021). Culturally, ‘Zhonghong’ poplar is a strong and admired tree species that is not only tolerant to drought, flooding, frost and temperatures as low as -33°C but also extremely adaptable. Moreover, it has the characteristics of high pest and pathogen resistance, easy management and a well-developed root system. It is suitable for planting in the Huang-Huai area, as well as in the northern part of China, and has prospects for very broad application in the greening of roadways, in urban and rural beautification and in urban plantings (Wang et al., 2021). This poplar cultivar has good ornamental characteristics and is fast-growing and productive (Sannigrahi et al., 2010). Plants with colorful leaves are important in urban landscaping and ecological tourism. In recent years, there have been many studies on the color mechanisms, photosynthetic characteristics, cultivation and propagation of such plants. A large number of studies have shown that the content and ratio of pigments such as chlorophyll, carotenoids and anthocyanins play an important role in the leaf coloration of plants that are valued for their foliage (Li et al., 2018; Chen et al., 2019). In poplar, researchers have identified several anthocyanins, including cyanidin, cyanidin 3-O-glucoside, delphinidin 3-O-glucoside and delphinidin 3-O-sambubioside (Alcalde-Eon et al., 2016). Many studies on the mechanism of leaf color transition have explored the relationship between leaf color change and changes in the content of each pigment in leaves and have identified some candidate genes related to anthocyanin synthesis (Mushtaq et al., 2016; Bao et al., 2022; Huang et al., 2022). ‘Zhonghong’ poplar is not only an internationally planted red-leaved poplar cultivar but also a colorful leafy ornamental and timber variety selected independently in China (Chen et al., 2022; Wang et al., 2022). However, there have been few studies on the mechanism of color presentation in ‘Zhonghong’ poplar, and there have been no reports on the differences in metabolites during the process of leaf color change. In this study, we investigated the variability of leaf brightness, color saturation, color phase change and metabolites during the transitions of different maturity stages and the metabolite changes in the most obvious period of leaf change after leaf expansion by using a broad targeted metabolomic analysis technique. We hypothesized that the red leaf color of ‘Zhonghong’ poplar is different from that of *Populus balsamifera*, and the main pigments of the red leaves in ‘Zhonghong’ poplar were inferred. The findings in this study reveal the changes in flavonoids during the process of leaf color change and provide a theoretical basis for understanding the coloration mechanism of ‘Zhonghong’ poplar.

## 2 Materials and methods

### 2.1 Study area

Mengzhou City, Henan Province (34°90′08″N, 112°69′66″E), is the location of the state-owned Mengzhou Forestry Farm on the south side of the town Xi Guo. The area has a warm temperate continental monsoon climate with four distinct seasons and an annual average temperature of approximately 14°C. The average annual precipitation is approximately 650 mm and the frost-free period is approximately 210 d. Annually, there are approximately 2160 h of sunshine and the dominant wind direction is from the northeast.

### 2.2 Plant materials and sampling

Three well-growing ‘Zhonghong’ poplar plants were selected in April 2019 and were sampled on April 1, April 6, and April 11, with the samples from these dates numbered R1, R2, and R3, respectively. The plants were sampled by selecting one red-leaved branch from the same location on each individual plant, with each individual plant serving as a biological replicate, and with three leaves sampled per branch. The leaves were placed in ice chests, brought back to the laboratory for immediate color phenotyping, and then quickly placed in liquid nitrogen.

### 2.3 Determination of the color phenotype

The *L* value (reflecting brightness, with positive indicating white and negative indicating black), a* value (reflecting the red/green phase, with positive indicating red and negative indicating green), and b* value (reflecting the yellow/blue phase, with positive indicating yellow and negative indicating blue) of fresh leaves were measured using a CR2500 colorimeter (MINOLTA, Japan). Ten locations on each leaf were randomly selected for measurement in a radiopaque environment (avoiding leaf veins), and the average of these 10 data points was ultimately taken as the final value of this index. Color intensity (C*) and color lightness values can be derived from the measured a*, b* and *L* values as follows.


$$C^{*}={\left(a^{*2}+b^{*2}\right)}^{\frac{1}{2}}$$

$$color\;lightness\;value=\frac{2000\cdot a^{*}}{L\cdot ({\text{a}}^{*2}+{\text{b}}^{*2{)}^{1/2}}}$$

### 2.4 Sample preparation and extraction

Freeze-dried leaves were crushed using a mixer mill (MM 400, Retsch) with a zirconia bead for 1.5 min at 30 Hz. Then, 100 mg of powder was weighed and extracted overnight at 4°C with 1.0 mL of 70% aqueous methanol. Following centrifugation at 10, 000 g for 10 min, the extracts were absorbed (CNWBOND Carbon-GCB SPE Cartridge, 250 mg, 3 mL; ANPEL, Shanghai, China, www.anpel.com.cn/cnw) and filtered (SCAA-104, 0.22 μm pore size; ANPEL, Shanghai, China, http://www.anpel.com.cn/) before LC-MS analysis.

### 2.5 HPLC conditions and ESI-Q TRAP-MS/MS

The sample extracts were analyzed using an LC-ESI-MS/MS system (HPLC, Shim-pack UFLC SHIMADZU CBM30A system, www.shimadzu.com.cn/; MS, Applied Biosystems 4500 Q TRAP, www.appliedbiosystems.com.cn/). The analytical conditions included HPLC: column, Waters ACQUITY UPLC HSS T3 C18 (1.8 µm, 2.1 mm*100 mm); solvent system, water (0.04% acetic acid): acetonitrile (0.04% acetic acid); gradient program,100:0 V/V at 0 min, 5:95 V/V at 11.0 min, 5:95 V/V at 12.0 min, 95:5 V/V at 12.1 min, 95:5 V/V at 15.0 min; flow rate, 0.40 mL/min; temperature, 40°C; and injection volume: 5 μL. The effluent was alternatively connected to an ESI-triple quadrupole-linear ion trap (Q TRAP)-MS.

LIT and triple quadrupole (QQQ) scans were performed on a triple quadrupole-linear ion trap mass spectrometer (Q TRAP) and API 4500 Q TRAP LC/MS/MS System equipped with an ESI Turbo Ion-Spray interface, operating in positive ion mode and controlled by Analyst 1.6.3 software (AB Sciex). The ESI source operation parameters were as follows: ion source, turbo spray; source temperature, 550°C; ion spray voltage (IS), 5500 V; ion source gas I (GSI), gas II (GSII), and curtain gas (CUR) at 55, 60, and 25.0 psi, respectively; and collision gas (CAD) pressure, high. Instrument tuning and mass calibration were performed with 10 and 100 μmol/L polypropylene glycol solutions in QQQ and LIT modes, respectively. QQQ scans were performed as MRM experiments with the collision gas (nitrogen) pressure set to 5 psi. DP and CE for individual MRM transitions were carried out with further DP and CE optimization. A specific set of MRM transitions were monitored for each period according to the metabolites eluted within this period.

### 2.6 Metabolome analysis

The extraction, detection and identification of metabolites were performed by Met Ware Biotechnology (Wuhan, China). The mass spectrometry data were processed with Analyst 1.6.3. Qualitative analysis was performed by the Metware in-house database (MWDB) and public metabolite information databases, and metabolite structural analysis was performed by referring to existing public mass spectrometry databases such as MassBank, KNApSAcK, Human Metabolome Database (HMDB) (Wishart et al., 2013), Metabolome Tomato Database (MoTo DB) and METLIN (Zhu et al., 2013). Metabolite quantification was performed using multi-reaction monitoring mode (MRM) of the triple four-level pole mass spectrum to analyze the data from different samples, peak area integration of the mass spectrum peaks of all substances was performed to ensure the accuracy of the qualitative quantification steps carried out to correct peak area integration (Fraga et al., 2010). The sample extracts were mixed and prepared as quality control samples (QC) for QC analysis. Overlapping presentation of total ion flow plots obtained from mass spectrometric detection of different QC samples allowed us to determine the reproducibility (technical replication) in the experimental operations.

Metabolomic data analysis included principal component analysis (PCA) (Eriksson et al., 2006), cluster analysis (HCA), difference fold analysis (fold change), orthogonal projections to latent structures-discriminant analysis (OPLS-DA) (Thévenot et al., 2015) differential metabolite screening and differential metabolite Kyoto Encyclopedia of Genes and Genomes (KEGG) functional annotation and enrichment analysis (Kanehisa and Goto, 2000). Differentially expressed metabolites were screened by the variable importance in projection (VIP) values of the OPLS-DA model combined with differential multiplicity values with the following criteria. 1) Metabolites with a fold change ≥ 2 or ≤ 0.5 were selected. 2) On the basis of the above, metabolites with VIP ≥ 1 were selected. The differences were considered significant.

### 2.7 Determination of pigment content

The photosynthetic pigment content of the samples was determined by the leaching method. The leaves were washed and ground (leaf veins were removed), and 0.1 g of ground leaf matter were weighed out (accurate to 0.01 g). Chlorophyll was extracted by adding 10 ml of 80% acetone and macerating the leaf matter in the dark for 72 h. The supernatant was then extracted. The anthocyanin was extracted by grinding leaf samples with 10 ml of 10% HCl methanol solution and then allowing the samples to rest for 4 h. The supernatant was then extracted by centrifugation at 3500 ×g for 15 min. The absorbance values of the chlorophyll extract at 445 nm, 644 nm, 662 nm and the anthocyanin extract at 530 nm and 657 nm were measured by a UV spectrophotometer (JINGHUA Instruments 752), and three biological replicates and three technical replicates were tested for each sample. The anthocyanin content (*C_A_*), chlorophyll a content (*C_a_*), chlorophyll b content (*C_b_*), total chlorophyll content (*C_T_*), and carotenoid content (*C_car_*) were calculated according to the formula presented in (Nesumi et al., 2012).


$$Chlorophy\mathrm{ll}\;a\;content\;\left(mg\times g^{-1}\right):\;C_{a}=\left(9.78\times A_{662}-0.99\times A_{644}\right)V/\left(W\times 1000\right)$$

$$Chlorophyll\;b\;content(mg\times g^{-1}):\;C_{b}=(21.40\times A_{644}-4.65\times A_{662})V/(W\times 1000)$$

$$Total\;chlorophyll\;content\;\left(mg\times g^{-1}\right):\;C_{T}=C_{a}+C_{b}$$

$$Carotenoid\;content\;(mg\times g^{-1}):C_{Car}=(4.69\times A_{445})\;V/(W\times 1000)-0.27\times (C_{a}+C_{b})$$

$$Anthocyanin\;content\;(mg\times g^{-1}):\;C_{A}=(A_{530}-0.25\times A_{657})V/(W\times 1000)$$

### 2.8 Data analysis

Mass spectrometry data were processed using Analyst 1.6.3 software. Statistical data were counted and plotted using Microsoft 365 Excel software.

## 3 Results and analysis

### 3.1 Leaf color variation

As shown in **Table 1**, In addition to differences in yellowing (b* value), there were significant differences in brightness (*L* value), reddening (a* value), color saturation (C* value), color light value and pigment content among the samples from the three sampling periods (**Table 1**). Across the sampling periods, the L value of the leaves increased from 22.94 to 31.32, with a change of 36.01%, and the a* value decreased from 17.06 to -1.52, with a change of 108.91%. When a* became negative, the leaves lost redness and transitioned to green; the b* value increased from 7.96 to 9.07, with a change of 13.94%; the C* value decreased from 18.47 to 8.85, with a change of 52.08%; and the color light value decreased from 84.13 to -11.22, with a change of 113.34%. In particular, the reddening and color light values changed most obviously; both were positive before April 6 but became negative on April 11, indicating that the leaves changed from red to green; the anthocyanin content decreased from 1.38 to 0.25, with a change of 81.88%; the chlorophyll a content increased from 0.45 to 0.87, with a change of 93.33%; the chlorophyll b content increased from 0.17 to 0.31, with a change of 82.35%; the total chlorophyll content increased from 0.87 to 1.48, with a change of 70.11%; and the carotenoid content increased from 0.25 to 0.31, with a change of 24.0%. The carotenoid content in the leaves was low, and the change over the sampling period was small. According to the analysis of the leaf pigments, the main substance responsible for the red coloration of ‘Zhonghong’ poplar was anthocyanin, and the phenomenon of the leaves changing from red to green was due to the sharp decrease in anthocyanin content and the increase in chlorophyll content.

**Table 1:** Analysis of changes in the leaf indexes of ‘Zhonghong’ poplar during different periods.

	Index	April 1	April 6	April 11
Leaf color parameters	Brightness *L*	22.94 ± 1.5b	27.17 ± 3.04a	31.32 ± 0.75a
	Redness a*	13.41 ± 4.2a	5.67 ± 3.3b	-2.45 ± 1.11c
	Yellowing degree b*	8.3 ± 1.01a	7.85 ± 1.31a	9.64 ± 1.07a
	Colour saturation C*	16 ± 3.39a	10.35 ± 1.46b	10.02 ± 1.19b
	Colour lightness value	71.99 ± 2.45a	42.89 ± 5.78a	-15.2 ± 2.26b
Pigment content	Anthocyanin	1.38 ± 0.01a	0.43 ± 0.00b	0.25 ± 0.00c
	Chlorophyll a	0.45 ± 0.02b	0.89 ± 0.02a	0.87 ± 0.02a
	Chlorophyll b	0.17 ± 0.01b	0.30 ± 0.01a	0.31 ± 0.01a
	Chlorophyll	0.87 ± 0.04b	1.51 ± 0.03a	1.48 ± 0.05a
	Carotenoids	0.25 ± 0.02b	0.31 ± 0.01a	0.31 ± 0.02a

Different lowercase letters: Significant differences (P<0.05)

The correlation coefficients of leaf color parameters and pigment content are shown in **Table 2**. Correlation analysis revealed that anthocyanin had a highly significant negative correlation with chlorophyll content, and that chlorophyll a and b content had a significant negative correlation with a*, while anthocyanin content had a highly significant positive correlation with a*, C* and color light value, indicating that over time, anthocyanin content decreased, while chlorophyll content gradually increased, and that the two changes were in opposite directions. The decrease in anthocyanin content caused the red leaf coloration to diminish. Chlorophyll a had a significant negative correlation with a* and C*, chlorophyll b content had a significant negative correlation with a*, C* and color light value, and the increase in chlorophyll content weakened the red coloration, color degree and color light value.

**Table 2:** Correlation analysis of the leaf color index and pigment content of ‘Zhonghong’ poplar during different periods (n=10).

	a*	b*	C*	Colour lightness value	*C_a_*	*C_b_*	*C_car_*	*C_A_*
*L*	-0.943** (<0.001)	0.660 (0.053)	-0.741* (0.022)	-0.969** (<0.001)	0.701* (0.035)	0.741* (0.022)	0.538 (0.135)	-0.802** (0.009)
a*	—	-0.519 (0.153)	0.848** (0.004)	0.962** (<0.001)	-0.720* (0.029)	-0.760* (0.017)	-0.564 (0.114)	0.832** (0.005)
b*	—	—	-0.119 (0.761)	-0.667* (0.050)	0.115 (0.769)	0.169 (0.663)	-0.105 (0.787)	-0.229 (0.554)
C*	—	—	—	0.709* (0.033)	-0.742* (0.022)	-0.739* (0.023)	-0.645 (0.061)	0.772* (0.015)
Colour lightness value	—	—	—	—	-0.627 (0.071)	-0.682* (0.043)	-0.472 (0.200)	0.760* (0.018)
*C_a_*	—	—	—	—	—	0.995** (<0.001)	0.929** (<0.001)	-0.977** (<0.001)
*C_b_*	—	—	—	—	—	—	0.924** (<0.001)	-0.991** (<0.001)
*C_car_*	—	—	—	—	—	—	—	-0.894** (0.001)

The data in the table are for the correlation coefficient, and the significance probability is included in parentheses. *: P<0. 05; **: P<0. 01.

As seen in **Table 2**, anthocyanin in the leaves of ‘Zhonghong’ poplar is the pigment that had the largest correlation coefficients with leaf color phase a* values, with C* and color light values of 0.832 (0.005), 0.772 (0.015) and 0.760 (0.018) at the three sampling periods, respectively. The correlation analysis revealed that anthocyanin content had a highly significant negative correlation with chlorophyll and carotenoid content. This indicates that anthocyanin is the main pigment affecting the reddening of ‘Zhonghong’ poplar leaves.

### 3.2 Evaluation of data

**Figure 1:**
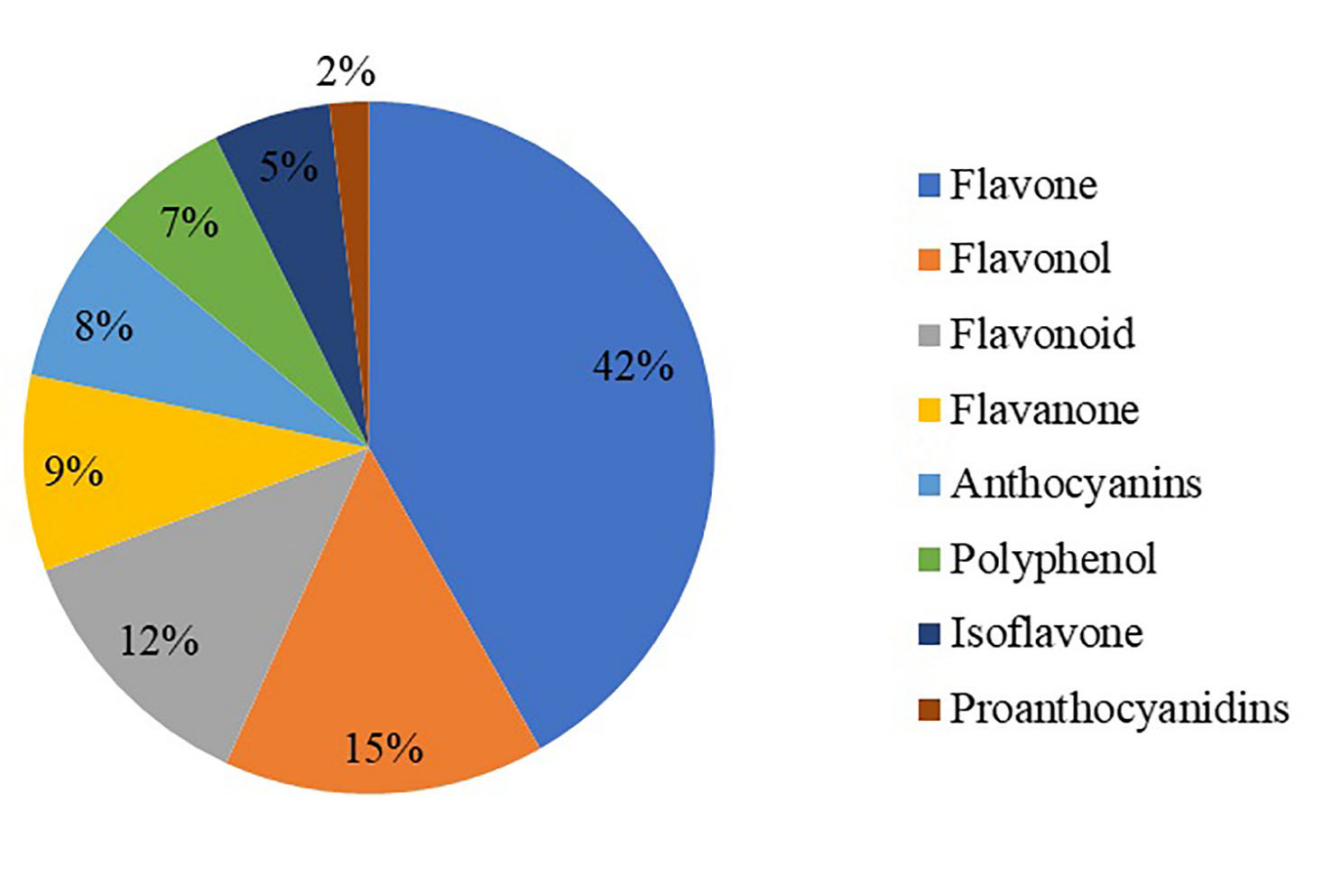
Statistical chart of flavonoid metabolite classification.

The metabolites of the samples were analyzed qualitatively and quantitatively by mass spectrometry based on the metabolic analysis by the flavonoid metabolome technology platform. Meanwhile, the base peak chromatograms were identified according to the above method, and a total of 273 flavonoid metabolites were identified, mainly in eight categories: polyphenols, anthocyanins, flavones, flavanols, flavonoids, flavanones, isoflavones, and proanthocyanidins (**Table S1**). Among these metabolites, flavones and flavonols were the most abundant, with 114 and 41 species, respectively, followed by flavonoids (34 species), flavanones (25 species), anthocyanins (21 species), polyphenols (18 species), isoflavones (15 species) and five proanthocyanidins (**Figure 1**).

**Figure 2:**
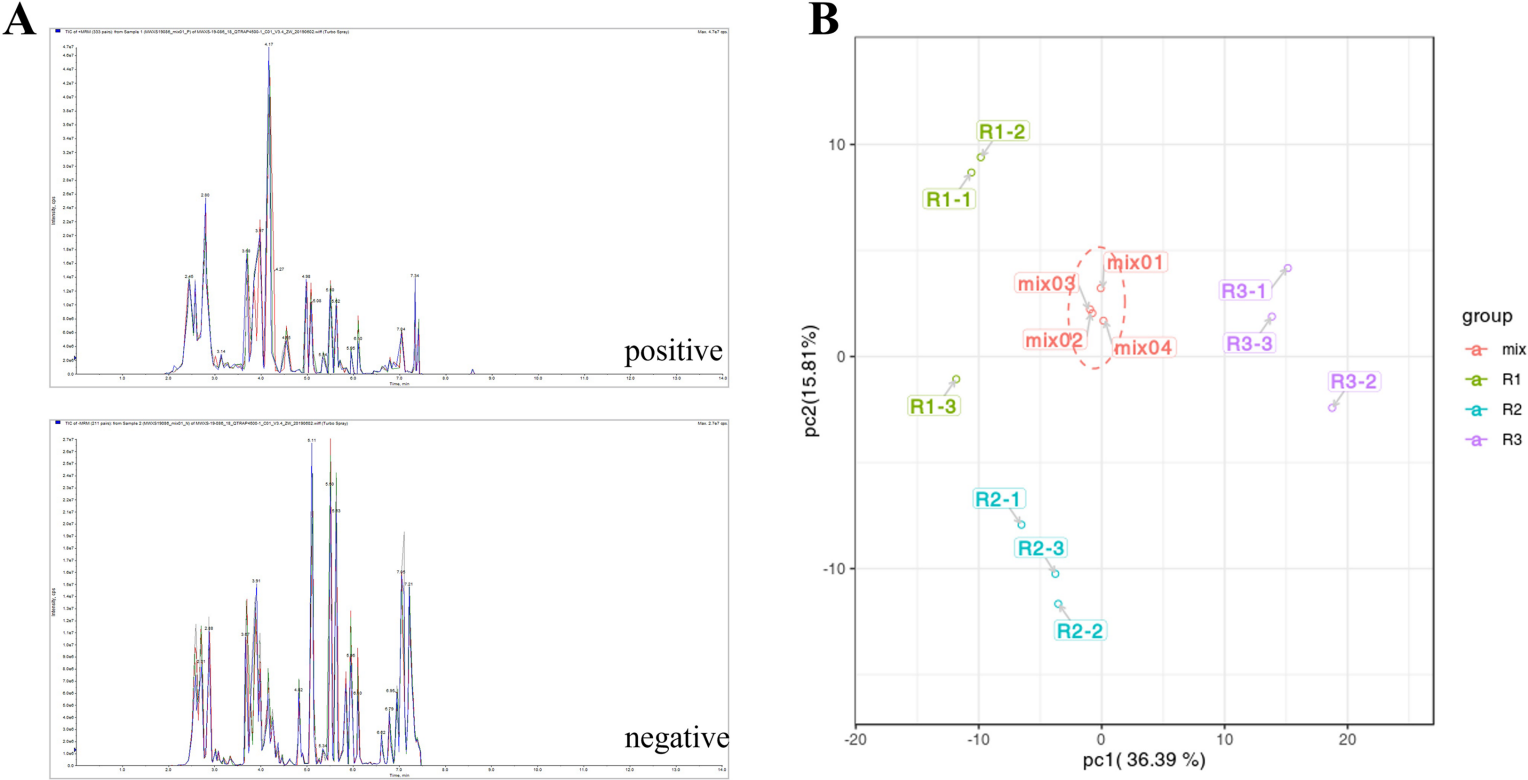
**(A)** QC-like intrinsic spectrum detection TIC overlay plot. (Negative and positive indicate the ion mode for mass spectrometry detection.) **(B)**: PCA score plot of quality spectrometry data for actual samples and quality control samples in each group. (The X axis represents the first principal component, and the Y axis represents the second principal component.).

The stability of metabolite extraction and detection results was determined by overlap analysis of the total ion flow (TIC) plots of the mass spectrometric detection of the different quality control samples for this experiment (**Figure 2A**). The results of TIC overlap analysis showed that the curves of total ion flow of all metabolites were highly overlapping, and the retention times and peak intensities were in obvious agreement, indicating that the metabolite extraction and samples had good stability in the mass spectrometric detection at different times, which was important for validating the subsequent experiments.

**Figure 3:**
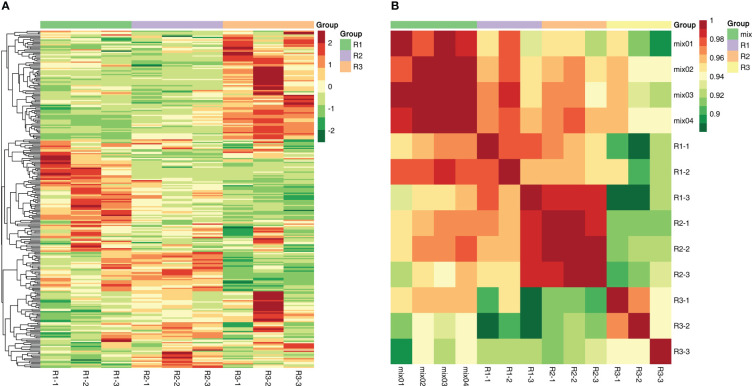
**(A)** The sample-to-metabolite correlation clustering diagram. **(B)**: The sample-to-sample correlation clustering diagram.

The scatter plot of the distribution of the corresponding samples was plotted by principal component analysis (PCA) (**Figure 2B**) to clarify the magnitude of variability between and within groups of samples. The PCA plots showed that there were large differences in metabolite components between different developmental periods of the same species, while the differences between different biological replicates from the same sample date were small. The first principal component, second principal component and third principal component accounted for 36.39%, 15.81% and 13.9% of the total variation, respectively (**Figure S1**). After normalizing the data, correlation analysis and clustering heatmap analysis between the overall metabolites and samples were performed, and a correlation clustering heatmap was generated (**Figure 3A**). The overall metabolite clustering of the samples showed that the repeats were clustered together during the same period, and the correlation analysis between the samples showed high correlation coefficients between samples of the same species in different periods, reflecting the large difference in metabolites among different kinds of leaves. In addition, correlation coefficients >0.9 between stages and >0.94 for the same stages (**Figure 3B**) indicate that overall, the samples were reproducible and that the obtained differentially expressed metabolite results were reliable.

### 3.3 Fold change analysis and screening for differentially expressed metabolites

**Figure 4:**
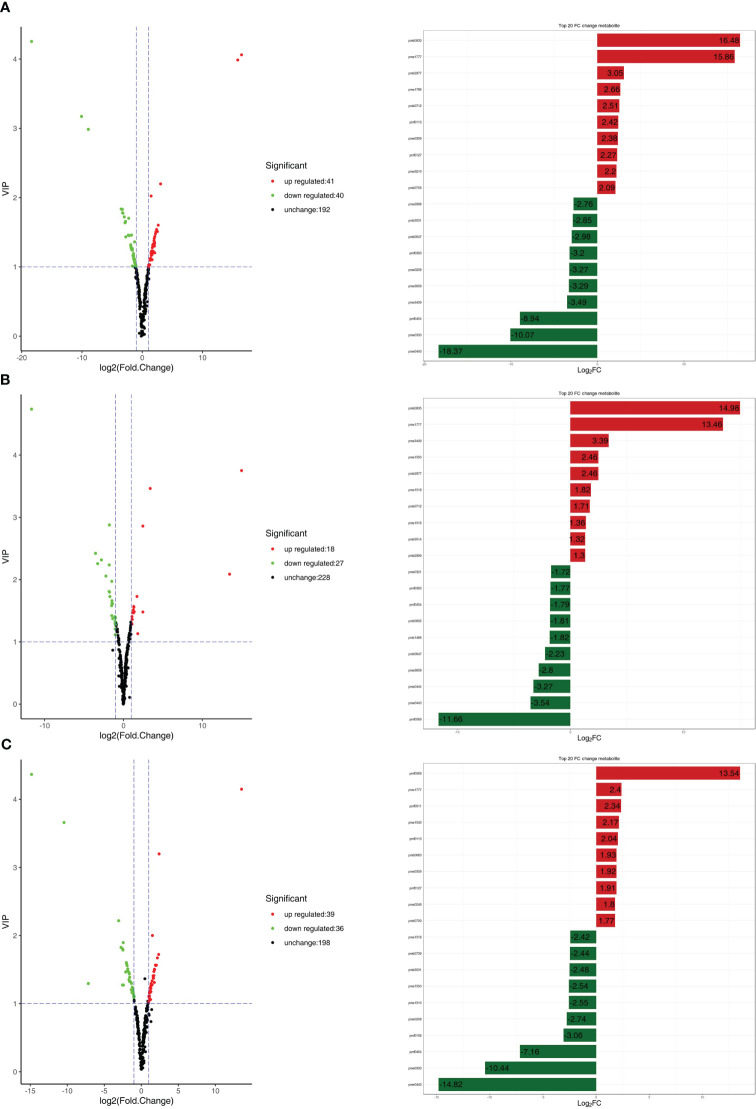
**(A)** On the right are the volcano plots of different metabolites. On the right are the top 10 upregulated and downregulated metabolites based on log2FC in the fold change analysis. **(A)** R1 vs. R3; **(B)** R1 vs. R2; **(C)** R2 vs. R3.

A large number of differentially expressed metabolites were detected in all comparison combinations. Eighty-one differentially expressed metabolites were detected in the R1 vs. R3 comparison, including 41 upregulated differentially expressed metabolites (49.38%) and 40 downregulated differentially expressed metabolites (50.62%); the most differentially paired upregulated metabolite was gallocatechin-gallocatechin at 16.48; and the most differentially paired downregulated metabolite was malvidin 3-O-galactoside, reaching -18.37 (**Figure 4A**). Forty-five differentially expressed metabolites were detected in the R1 vs. R2 comparison, including 18 upregulated differentially expressed metabolites (60.00%) and 27 downregulated differentially expressed metabolites (40.00%). The most differentially paired upregulated metabolite was gallocatechin-gallocatechin, reaching 14.98. The most differentially paired downregulated metabolite was liquiritin, reaching -11.66 (**Figure 4B**). Seventy-five differentially expressed metabolites were detected in the R2 vs. R3 comparison, including 39 upregulated differentially expressed metabolites (48.00%) and 36 downregulated differentially expressed metabolites (52.00%). The metabolite with elevated expression showing the largest fold change was liquiritin, which reached 13.54. The most downregulated metabolite with the highest differential fold change was malvidin 3-O-galactoside, with a fold change of -14.82 (**Figure 4C**). This indicates that the metabolite differences between the second and third periods during leaf development were significantly greater than those between the first and second periods, indicating that the differential metabolite types were relatively conserved from the first to the second periods. Most of the differentially expressed metabolites showed a downregulation trend in the second period and an upregulation trend in the third period, i.e., the differential metabolite content showed a trend of first decreasing and then increasing (**Table S2**).

**Figure 5:**
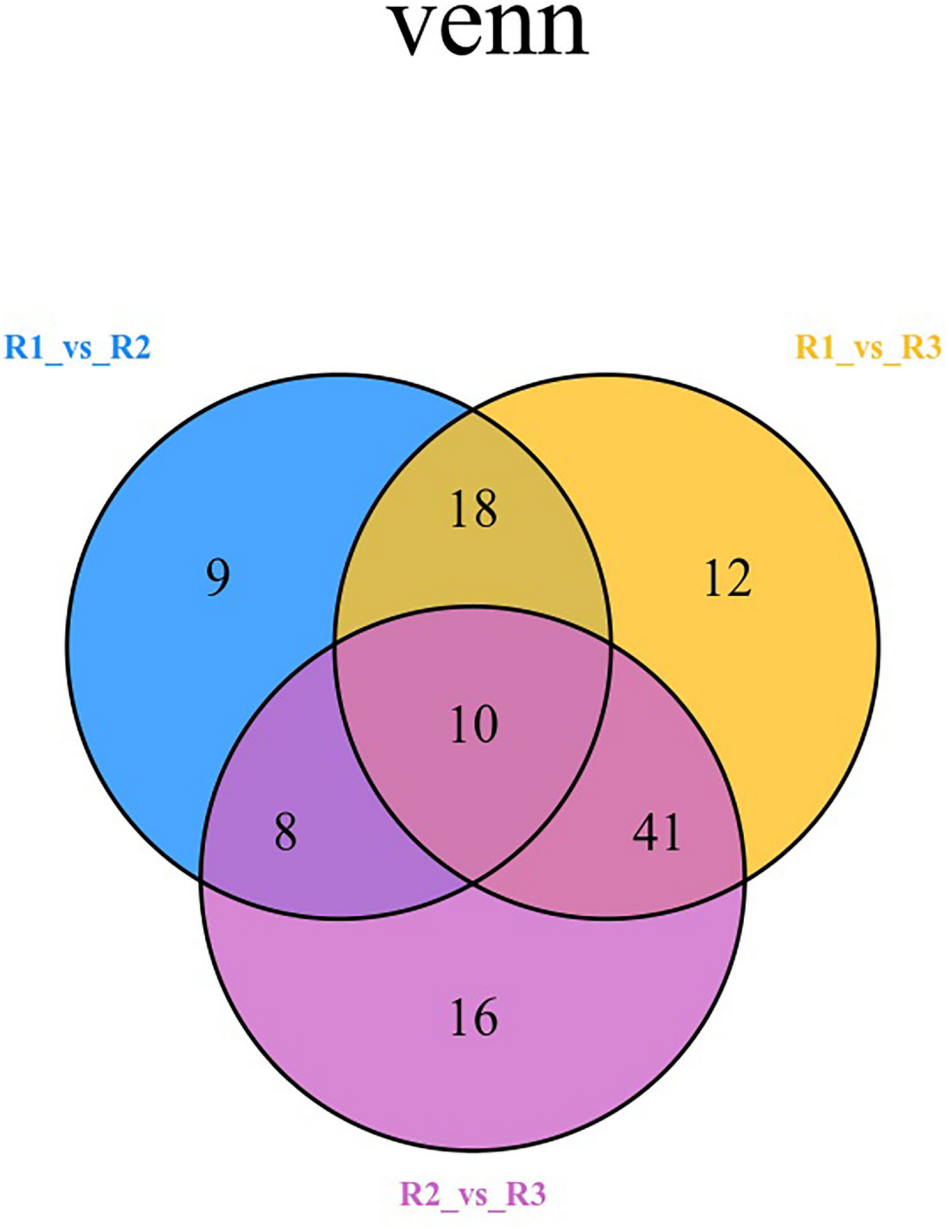
Venn diagram comparing different metabolites in different periods.

The Venn diagram shows the changes in differentially expressed metabolites in different periods of red leaf growth and development, from which it can be seen that R1 vs. R2 has 9 characteristic differential metabolites, R1 vs. R3 has 12 characteristic differential metabolites, and R2 vs. R3 has 16 characteristic differential metabolites (**Figure 5**; **Table S3**). The metabolites that showed significant differences in R1 vs. R2, R1 vs. R3, and R2 vs. R3 were 10 in number, and the metabolites that occupied the largest proportion were flavonoids, with 4 metabolites, namely, anhydroglycinol, persicogenin, silibinin and liquiritin. Additionally, 2 anthocyanins were included, as well as 1 polyphenol, 1 flavanone, 1 isoflavone, and 1 flavone. These 10 metabolites were differentially expressed in the three comparative combinations and were presumed to be the “core metabolome” for leaf color changes in the red leaves of ‘Zhonghong’ poplar during different developmental periods (**Table 3**).

**Table 3:** Metabolite statistics of the “core metabolome”.

Compounds	Class	Compound ID	Pathway ID
Di-gallocatechin	Polyphenols	–	–
Mallow pigment 3-O-galactoside	Anthocyanin	–	–
Phloroglucin	Anthocyanin	C08639	ko00942
Purple rivastigmine	Flavanones	C08578	ko00941
Ichthyone	Isoflavone	C07593	ko00943
Apigenin-6,8-di-C-glucoside	Flavonoid	–	–
Anhydroglycinol	Flavonoids	C10200	–
Peach glycosides	Flavonoids	–	–
Silymarin	Flavonoids	C07610	–
Glycyrrhizin	Flavonoids	C16978	–

–: The metabolite was not annotated to a pathway.

### 3.4 KEGG functional annotation and enrichment analysis of differentially expressed metabolites

**Figure 6:**
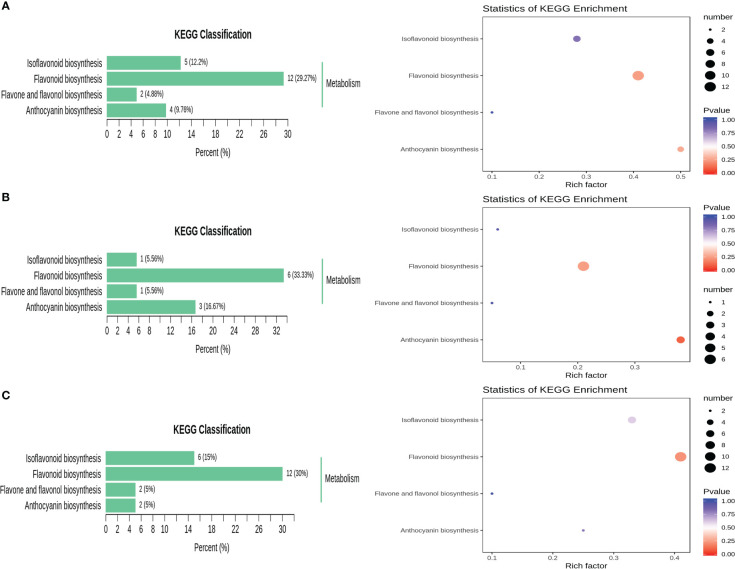
KEGG classification map and KEGG pathway enrichment of different metabolites. **(A)** R1 vs. R3; **(B)** R1 vs. R2; **(C)** R2 vs. R3.

The detected metabolites were annotated using the KEGG database, and the annotation results were subsequently classified according to the KEGG pathway types. The results in **Figure 6A** show that the differentially expressed metabolites were mainly enriched in the following four metabolic pathways: flavonoid and flavonol biosynthesis (ko00944), flavonoid biosynthesis (ko00941), isoflavone biosynthesis (ko00943) and anthocyanin biosynthesis (ko00942). The differentially abundant metabolites were mainly enriched in the flavonoid biosynthetic pathway at different developmental periods. Secondly, they were enriched in the anthocyanin biosynthetic pathway in R1 VS R2 and in the isoflavone biosynthetic pathway in R1 vs. R3 and R2 vs. R3. KEGG enrichment analysis showed that the pathway in which the differentially expressed metabolites in R1 vs. R3 and R1 vs. R2 were most significantly enriched was the isoflavone biosynthetic pathway, and the pathways in which the differentially expressed metabolites in R2 vs. R3 were most significantly enriched were the flavonoid and flavonol biosynthetic pathways (**Figures 6B, C**).

**Figure 7:**
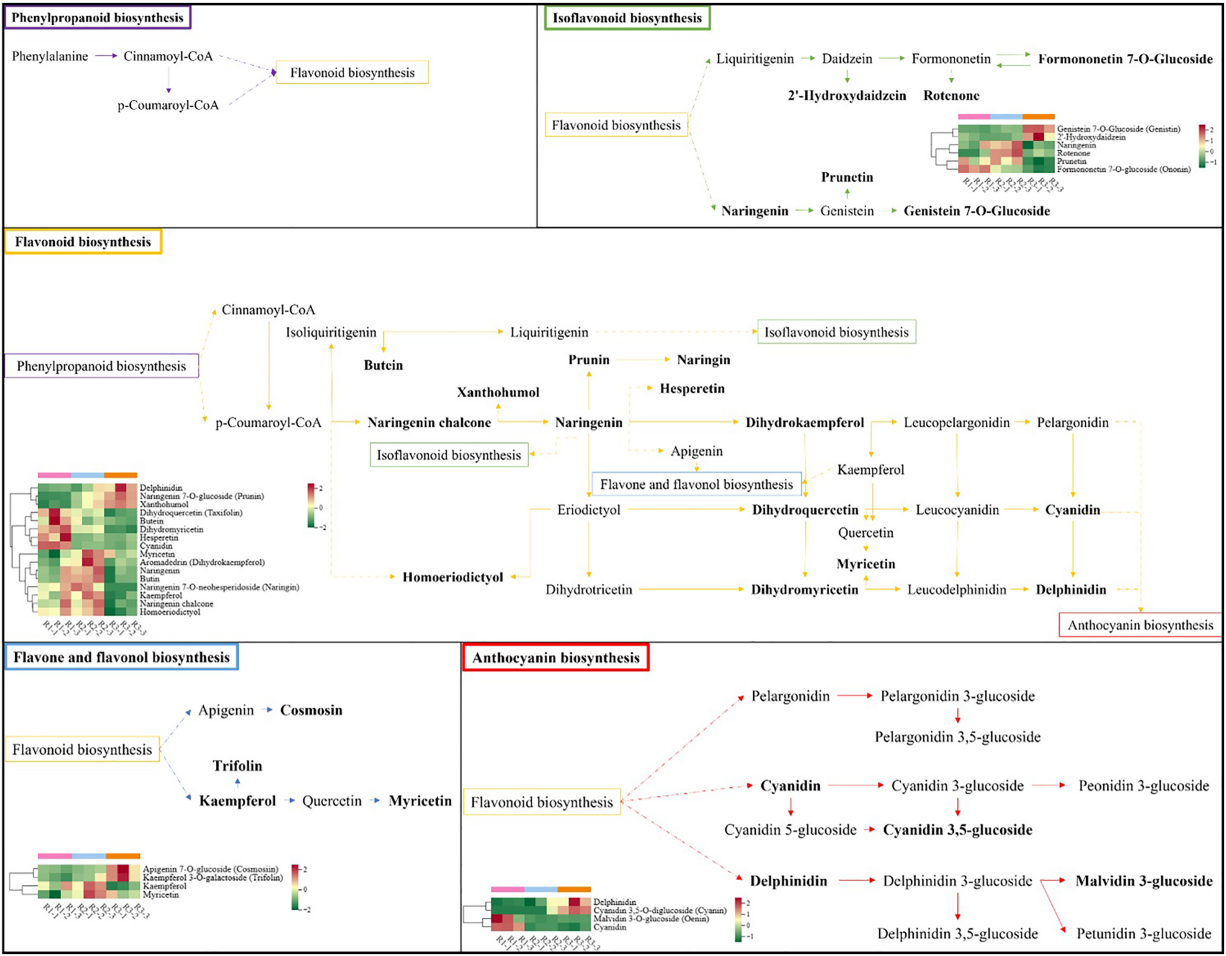
Schematic diagram of the pathways involved in the synthesis of flavonoid metabolites. Each heatmap shows metabolites with significantly different expression levels.

The participation of significantly different metabolites in the metabolic pathway in each comparison combination is shown in **Table S4**. Four metabolites were enriched in R1 vs. R3, with upregulated expression of delphinidin and cyanidin 3,5-O-diglucoside (cyanin) and downregulated expression of malvidin 3-O-glucoside (oenin) and cyanidin; three metabolites were enriched in R1 vs. R2, with upregulated expression of cyanidin 3,5-O-diglucoside (cyanin) and downregulated expression of malvidin 3-O-glucoside (oenin) and cyanidin; two metabolites were enriched in R2 vs. R3, and both delphinidin and cyanidin 3,5-O-diglucoside (cyanin) were upregulated (**Figure 7**). These results suggest that the leaf color shift is associated with the continuous downregulation of malvidin 3-O-glucoside (oenin) and cyanidin.

## 4 Discussion

Plant leaf color is the result of a combination of genetic and environmental factors (Yan et al., 2022), but many influencing factors ultimately act on pigment function. The direct cause of the colorful appearance of certain colored plants is a change in the type and proportion of pigments in the leaves (Lu et al., 2020a). Not only has the content of anthocyanin been found to be significantly higher in purple kale leaves than in green kale leaves (Ren et al., 2019), but it has been confirmed in several species with colored leaves that anthocyanin is the direct cause of the red coloration (Chen et al., 2021). Our results showed that the leaves showed a purplish-red color, coinciding with the highest percentage of total anthocyanin content, in the first stage and a brownish-green color, coinciding with the highest percentage of chlorophyll content, in the third stage. The most important factor for the red color of the leaves was that the leaves contained higher anthocyanin and lower chlorophyll. When the anthocyanin content decreases and the chlorophyll content increases, the leaf color changes from purple-red to brown−green, further indicating the degree of influence of the difference in total anthocyanin content on the color change in ‘Zhonghong’ poplar leaves. We speculate that in the early stage of leaf maturation, anthocyanin, as a common functional pigment substance in plants, accumulates in large amounts to protect leaves from UV light. As the leaves mature, leaf antibodies are enhanced to meet the plant growth requirements, a large amount of chlorophyll is synthesized, and the accumulation of anthocyanin is reduced. It has been reported that the reduction in anthocyanin content may occur as a result of degradation of anthocyanin and termination of anthocyanin synthesis, or it may be accompanied by leaf maturation, leaf expansion, and dilution of anthocyanin in the tissues. However, whether this biological process is closely related to the degradation of anthocyanins requires further investigation.

Anthocyanins are an important class of water-soluble pigments, with 6 common types: pelargonidin (Pg), cyanidin (Cy), delphinidin (Dp), peonidin (Pn), petunidin (Pt), and malvidin (Mv) (Chen et al., 2023). Pelargonidin is associated with a brick-red color, cyanidin and peonidin lead to a purplish-red color, and delphinidin, petunidin, and malvidin cause a blue−purple color (Hughes and Lev-Yadun, 2015; Xia et al., 2022). Anthocyanins contribute directly to leaf color, and other nonanthocyanin polyphenols also influence leaf coloration through auxin effects, with flavanols and flavones having the most significant effect on flower and leaf color (Song et al., 2017). Thwe measured anthocyanins in buckwheat hairy root material and hypothesized that delphinium pigments and their derivatives are not only responsible for blue hues but may also contribute to red coloration (Thwe et al., 2014). We found an important scabiolide derivative, scabiolide 3-O-glucoside, which was the most highly expressed flavonoid metabolite in all three periods of red leaves, and therefore, it is likely the most important metabolite responsible for the red coloration of ‘Zhonghong’ poplar leaves. This result is aligned with the results of similar studies, such as that of Ni et al. (Ni et al., 2018), who applied UPLC−MS to detect eight anthocyanidins in the red pericarp of apricot trees and found that centaureidin 3-O-glucoside was the predominant color-presenting pigment in the red pericarp. Abdel-Aal et al. also detected significant amounts of centaureidin 3-O-glucoside in purple-grained wheat (Abdel-Aal et al., 2018). Of course, we were more interested in why the leaf color changed. Therefore, we analyzed the changes in the content of these flavonoid metabolites at three leaf developmental stages. Naringenin was found to be significantly downregulated in expression, which directly affected the formation of the precursors of anthocyanin synthesis, dihydromyricetin (DHK), dihydromyricetin (DHM), and dihydroquercetin (DHQ). Dihydroflavanols (DHK, DHM, DHQ) are catalyzed by DFR to produce the corresponding colorless anthocyanins (progeranosides, proferulosides, and procornflowerosides), which are then catalyzed by colorless anthocyanin dioxygenase/anthocyanin synthase (LDOX/ANS) to form colored anthocyanins. This explains why the decrease in naringin content as the leaves mature directly affects the synthesis of anthocyanins.

In our study, small amounts of dihydromyricetin (DHK) and dihydromyricetin (DHM) could be detected, but the presence of dihydroquercetin (DHQ) was not detected. The combination of the detected centaureidin and a large number of centaureidin derivatives, such as centaureidin 3-O-glucoside, suggests a complete reaction of dihydroquercetin (DHQ). For dihydromyricetin (DHK), the only geranoside, a florigenic glycoside product, was detected with low expression; presumably, this class of pigments has little effect on the color presentation of ‘Zhonghong’ poplar leaves. For dihydromyricetin (DHM), a large number of downstream products were detected, and the response was relatively complete. Among them, special attention was given to the mallow pigment 3-O-glucoside in the delphinium branch and scabiolide on the scabiolide branch, which showed a significant downregulation trend at all three stages and were the main metabolites responsible for the change in leaf color from purple−red to brown−green in ‘Zhonghong’ poplar. Meanwhile, we found that gallocatechin was significantly upregulated in expression with leaf development. Gallocatechin and epigallocatechin are the main colorless polyphenols in ‘Zhonghong’ poplar leaves and are not directly involved in leaf color development but play a more important role in the anthocyanin synthesis pathway. According to the anthocyanin synthesis pathway, LAR catalyzes the synthesis of the corresponding flavonoid products (aflatoxin, gallocatechin and catechin) from proanthocyanidin glycosides, and ANR reduces anthocyanin glycosides and synthesizes the corresponding flavonoid products (epiflatoxin, epigallocatechin and epicatechin). As mentioned above, there is clearly a reciprocal relationship between anthocyanin and flavonoid products, so the synthesis of gallocatechin in large quantities will inevitably affect the synthesis of mallow in glycoside derivatives. Lu Xiaoyu found that the overexpression of LAR and ANR genes leads to an increase in the content of proanthocyanins and a decrease in the content of anthocyanins, which was also confirmed by Liu (Liu et al., 2013; Lu et al., 2020b). In tea plant, Pan found that compared to green leaves, red leaves contained more catechins and epicatechins, and as the leaves grew, the catechin content showed a significant decreasing trend, while the epicatechin content did not change much (Pan et al., 2020). However, it has also been shown that the reduced content of polyphenols in purple or red-leaved plants is one of the reasons for leaf coloration (Ying et al., 2017). Nesumi has reported that the polyphenol content in purple tea leaves is lower than that in green tea leaves (Nesumi et al., 2012). It is evident that the effect of catechin and epicatechin content in different plants on the coloration of red and purple leaves is not entirely consistent. The detected substances can provide a reference for studying the pattern of variation in flavonoid substances and the development and utilization of the ‘Zhonghong’ cultivar of poplar.

## 5 Conclusion

In this paper, we investigated the leaf color parameters, the pigment content and their correlation, as well as the changes in metabolites associated with leaf color of ‘Zhonghong’ poplar by metabolomic analysis. Furthermore, we elucidated the coloring mechanism, from phenotype and pigment content to metabolites, of ‘Zhonghong’ poplar leaves in three sampling periods. We found that anthocyanin (*C_A_*) was the most important color parameter of the leaves and was significantly correlated with brightness (*L* value), reddening (a* value), chlorophyll a (*C_a_*), chlorophyll b (*C_b_*), and carotenoid (*C_car_*) content but not with the b* value. No significant correlations were found between a* and b*. Next, it was shown that ‘Zhonghong’ poplar leaves contained 273 flavonoid metabolites, mainly polyphenols, anthocyanins, flavones, flavanols, flavonoids, flavanones, isoflavones and proanthocyanidins. Among them, flavonoids and flavanols were the most diverse and occupied the largest proportion of the flavonoid metabolites. In our study, 114 flavonoids and 41 flavanols were detected. The results suggested that the shift of red leaves from a bright purplish red color to a brownish green color was associated with the continuous downregulation of malvidin 3-O-glucoside (oenin) and cyanidin. The detected substances can provide a reference for the study of the variation pattern in flavonoid substances and the development and utilization of the ‘Zhonghong’ poplar cultivar.

## Data availability statement

The datasets presented in this study can be found in online repositories. The names of the repository/repositories and accession number(s) can be found in the article/**Supplementary Material**.

## Author contributions

JZ and DL designed the study. MC completed the experimental part. CC and HL collected the data. LH analyzed the data. MC and CC wrote the manuscript. ZZ contributed to the illustration. All authors contributed to the article and approved the submitted version.
