# Supplementary figures and images for: Metabolome analysis reveals flavonoid changes during the leaf color transition in Populus × euramericana ‘Zhonghuahongye’
Source: Front Plant Sci. 2023 May 8;14:1162893. doi: 10.3389/fpls.2023.1162893 (PMC10200940; doi:10.3389/fpls.2023.1162893)

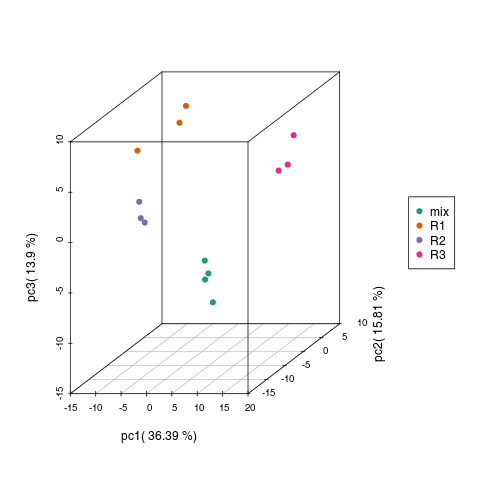

Supplement: Supplementary file 1 [file DataSheet_1.zip › Data Sheet 1 (13)/Supplementary Figures/Figure S1.png]
